# EFFECTS OF MIRROR THERAPY IN POST-TRAUMATIC COMPLEX REGIONAL PAIN SYNDROME TYPE-1: A RANDOMIZED CONTROLLED STUDY

**DOI:** 10.2340/jrm.v56.40417

**Published:** 2024-09-24

**Authors:** Elif Can ÖZDEMIR, Atilla H. ELHAN, Ayșe A. KÜÇÜKDEVECI

**Affiliations:** 1 Department of Physical Medicine and Rehabilitation, Gülhane Training and Research Hospital, Ankara; 2 Department of Biostatistics, Faculty of Medicine, Ankara University, Ankara; 3 Ankara University, Faculty of Medicine, Department of Physical Medicine and Rehabilitation, Ankara, Turkey

**Keywords:** mirror therapy, complex regional pain syndrome, CRPS, physical therapy, rehabilitation

## Abstract

**Objective:**

To investigate the effects of mirror therapy applied in addition to routine rehabilitation on clinical outcomes in post-traumatic complex regional pain syndrome type 1.

**Design:**

Single-blind randomized controlled trial.

**Subjects:**

Patients with trauma-induced complex regional pain syndrome type 1 of the hand receiving outpatient rehabilitation.

**Methods:**

Patients were randomized into mirror therapy and control groups. All patients received routine physical therapy and rehabilitation for 20 sessions (5 sessions/week, for 4 weeks). The mirror group received additional mirror therapy at each session. The primary outcome was pain intensity by numeric rating scale. Secondary outcomes were grip/pinch strength, hand/wrist circumference, dexterity, hand activities, and health-related quality of life. All assessments were performed before and immediately after the treatment, and 4 weeks later at follow-up.

**Results:**

Forty patients were enrolled, 20 in each group. Both groups revealed statistically significant improvements from therapy regarding pain, grip/pinch strength, wrist circumference, dexterity, and hand activities (*p* < 0.05). When groups were compared regarding the improvements in assessment parameters, no statistically significant difference was found between the 2 groups in any of the outcomes (*p* > 0.05).

**Conclusion:**

Mirror therapy applied in addition to routine therapy in post-traumatic complex regional pain syndrome type 1 did not provide extra benefit to the improvement of pain, function, and other clinical outcomes.

## LAY ABSTRACT

Complex regional pain syndrome type 1 (CRPS-1) is a chronic pain condition that often presents as a result of fracture, surgery, other traumatic factors, or stroke. Pain and functional impairment of the affected hand may cause limitations in daily activities. Rehabilitation is the first-line treatment for CRPS-1 and should be started as soon as possible together with analgesic treatment. Mirror therapy is a neurorehabilitative exercise and is used as complementary to other rehabilitation methods. Currently, its effects in post-traumatic CRPS-1 remain unknown. Therefore, this study investigated the effects of mirror therapy prescribed in addition to a conventional physical therapy and rehabilitation programme. Our results revealed that both “mirror plus routine therapy” and “routine therapy” groups improved with therapy regarding pain as well as hand strength, oedema, dexterity, and activities. However, mirror therapy applied in addition to routine therapy did not provide extra benefit to the improvement of pain, function, and other clinical outcomes.

Complex regional pain syndrome (CRPS) is a chronic pain condition, usually beginning in a distal extremity and characterized by inflammatory and autonomic features in the region of pain (1). It often presents following injury (most commonly fractures, but also sprains, contusions, and crush injuries), peripheral nerve damage, or surgery where the magnitude or duration of pain is disproportionate to the inciting event (1). CRPS type 1 (CRPS-1) is the clinical sub-type with no nerve injury. The pathogenesis of CRPS-1 is complex and multifactorial (2). Both peripheral and central mechanisms are involved (1). In the early stage of CRPS-1, proinflammatory cytokines increase, depolarization occurs in A-delta and C fibres, and neurogenic inflammation follows. Over time, maladaptive cortical changes occur in the central nervous system. According to most studies in the literature, the main cause of pain in CRPS is due to the incompatibility between visual and sensory inputs to the parietal cortex and the motor cortex, as in phantom pain (2). Pain is an important clinical problem that has negative effects on patient function. Management of CRPS-1 requires a multimodal approach. Rehabilitation, which consists of physical and occupational therapy combined with a relevant exercise programme, is the first-line treatment for CRPS-1 and should be started as soon as possible together with pharmacological analgesic treatment (3, 4).

Mirror therapy (MT) is a neurorehabilitative exercise used as complementary to other rehabilitation methods. Mirror therapy provides visual feedback as if there is movement in the affected hand (5). Visual and proprioceptive feedback alters neural plasticity in the brain, reducing pain and increasing the excitability of the motor cortex (6–8). Mirror therapy also acts on the mirror neuron system by promoting mirror neurons (9). Mirror neurons activate the corticospinal pathway, increasing motor learning and motor activity.

Recently, studies have revealed the positive effects of mirror therapy in patients diagnosed with CRPS-1. Among the published studies, there are 4 randomized controlled trials, all of which investigated the effects of mirror therapy in post-stroke CRPS-1 (10–13). Regarding the CRPS-1 that develops secondary to traumatic factors, there are only a few studies in the literature and they enrolled small and heterogeneous patient groups or did not assess pain and functionality (14, 15).

Therefore, this study was planned to investigate the effects of mirror therapy on pain and other clinical outcomes in trauma-induced CRPS-1. We hypothesized that mirror therapy prescribed in addition to a conventional physical therapy and rehabilitation programme would be more effective in reducing pain and improving function compared with only conventional treatment in patients with CRPS-1 of the hand developed secondary to traumatic factors.

## METHODS

### Study design and participant selection

This study was planned as a prospective, single-blind, randomized controlled clinical trial. Patients who were referred to the hand rehabilitation outpatient clinic at the Department of Physical Medicine and Rehabilitation, Ankara University Medical Faculty Hospitals with complaints of pain, swelling, or redness in their hands between March 2017 and April 2019 were evaluated and screened regarding the diagnosis of CRPS-1.

*Inclusion criteria*: patients aged ≥18 with a diagnosis of CRPS-1 according to 2003 Budapest diagnostic criteria (16), patients who developed CRPS-1 of the hand due to traumatic causes (surgical procedures, fractures, and/or immobilization of the hand and/or the upper extremity), patients who agreed to participate in the study and signed the informed consent form.

*Exclusion criteria*: CRPS type 2, post-stroke CRPS-1, patients who were in the acute and post-acute rehabilitation phase of tendon repair of the hand, comorbid conditions (e.g., decompensated heart failure, chronic renal insufficiency, malignancy) that would affect the functioning and health-related quality of life (hQoL) of the person, hand arthritis, acute deep arterial/vein thrombosis in the upper extremity, arterial/venous injuries and/or undergoing arterial revascularization, patients with excessive alcohol and inappropriate opioid use, untreated psychiatric disease, and recurrent CRPS-1.

The protocol was approved by the Ankara University Faculty of Medicine Clinical Research Ethics Committee (Decision No: 03-109-17, date: 2 February 2017) and the trial was executed in accordance with the Declaration of Helsinki and International Conference on Harmonization Good Clinical Practice Guidelines. The study was registered on Clinical Trials (Trial Id: NCT03377504).

### Interventions

Participants were allocated randomly into 2 groups: mirror and control. All patients received a routine physical therapy and rehabilitation programme for 20 sessions (5 sessions per week, 45–60 minutes a day, total 4 weeks) at the hand rehabilitation unit of the department. The mirror group received additional mirror therapy to the affected hand for 30 min/session.

During mirror therapy, the patient was seated in front of a 35x50 cm mirror placed vertically on the table. The affected arm of the patient was placed behind the mirror, while the unaffected hand was placed in front of the mirror. Patients were trained to perform various movements of the unaffected side: active flexion, extension, ulnar/radial deviation of the wrist; finger flexion, extension, abduction, adduction; thumb opposition; forearm supination, pronation as well as grasping activities of various objects such as pen, key, glass, ball, cube, etc. Simultaneously, the patient was asked to make the same movements with the affected hand as much as was possible.

The routine physical therapy and rehabilitation programme included physical modalities (contrast bath, TENS, hot pack to the affected hand), desensitization, exercises, and occupational therapy. After desensitization, passive, active-assisted, and active range of motion exercises, slow flexibility, and isometric strengthening exercises were started. When improvement was achieved, weight-bearing (stress loading) exercises were commenced and followed by isotonic strengthening (spring, ball, dough tightening exercises). Occupational therapy as part of the functional restoration process was administered to all patients. Within the scope of this therapy, coordination and dexterity exercises as well as activities for grasping different objects were exercised to ensure the use of the affected extremity in daily life. Training was given for daily living activities such as feeding, dressing, personal care, toilet use, and household activities. This protocol is routinely applied in our hand rehabilitation unit for CRPS patients (17) and was modified from the protocol suggested by Harden et al. (18).

As pharmacotherapy, all patients were prescribed oral acemetacin 60–120 mg/day for its anti-inflammatory effect during the 4-week treatment period.

### Outcome measures

All patients were assessed by the same physical medicine and rehabilitation (PMR) physician (ECÖ), who was blinded to the randomization and the treatment procedure. Evaluations were performed at baseline before the treatment, immediately after the treatment (week 4), and then 4 weeks later at follow-up (week 8).

The primary outcome measure was pain severity, which was assessed by a numeric rating scale (NRS) where scores ranged from 0 (no pain) to 10 (most severe pain) (19). Secondary outcome measures were grip strength, lateral pinch strength, hand/wrist circumference, hand dexterity, hand functioning in activities of daily living, and hQoL. A Jamar Dynamometer (OPC Health, Clayton, VIC, Australia) was used to measure grip strength. A pinch meter was used to measure lateral grip strength. All measurements were repeated 3 times and the highest value (in kg) was recorded.

For wrist circumference measurements, the tape measure was taken from the narrowest region, just proximal to the styloid processes of the radius and ulna. Hand circumference measurement was performed at the level of the 3^rd^ MCP joint. The average of the 3 consecutive measurements was taken. The Moberg pick-up test was used to evaluate hand dexterity (20). The time (in seconds) taken by the participant to pick up and put the 12 small metallic objects into the box was recorded as the score of the test. Hand functioning in activities of daily living was evaluated by the Duruöz Hand Index (DHI) (21), which includes 18 items. The total score ranges between 0 and 90, higher score indicating higher hand-related disability. The Nottingham Health Profile (NHP) subsections were used to assess the hQoL in terms of pain, sleep, emotional reactions, and social isolation (22). Each subsection is scored between 0 and 100, a higher score indicating worse health status.

### Sample size

The sample size was calculated based on the primary outcome variable, that is, ΔPain (change) score (0–10 NRS). Group sample sizes of 18 and 18 achieved 81% power to detect a difference of 1.0 in terms of ΔPain with estimated group standard deviations of 1.0 and 1.0 and with a significance level of 0.05 using a 2-sided Mann–Whitney *U* test. Considering that there might be a 10% dropout rate, the sample size was determined as 40 participants.

### Randomization/blinding

All participants were randomly allocated to 1 of 2 groups: mirror and control groups. A block randomization with a block size of 2 was used to ensure an equal number of subjects in each group. The randomization process and confidentiality were coordinated by the responsible physiotherapist (BÖ). In this single-blind study, all patients were evaluated by the same physician (ECÖ), and the evaluator was blinded to the patient’s treatment groups.

### Statistical analysis

Student’s *t*-test for normally distributed continuous variables, the Mann–Whitney *U* test for ordinal or non-normally distributed continuous variables, and the χ^2^ test or Fisher’s exact test for categorical variables were used for comparisons between 2 independent groups. The Wilcoxon signed-ranks test was used to evaluate differences for within-group comparisons. Bonferroni correction was applied for all possible multiple comparisons to control the Type I error rate. A *p*-value less than 0.05 was considered significant.

## RESULTS

**Fig. 1:**
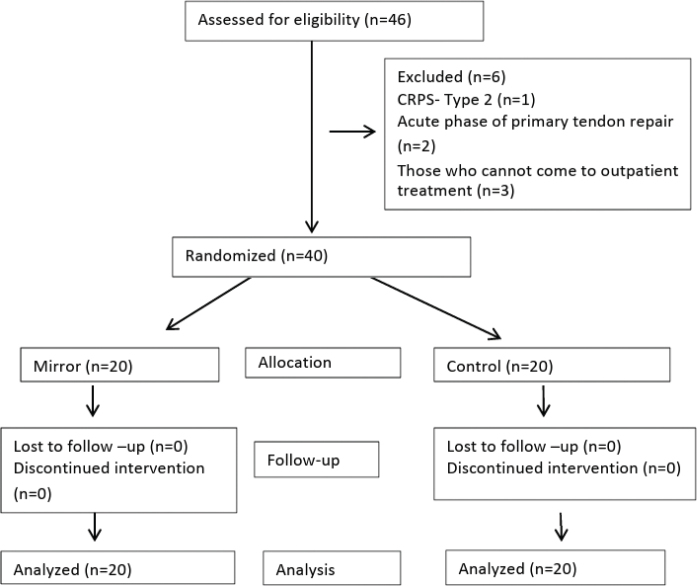
Study flowchart.

Forty-six patients were screened for eligibility between March 2017 and April 2019. Three patients did not meet the inclusion criteria and 3 patients declared that they could not attend the outpatient treatment sessions. Consequently, 40 patients were randomly assigned to 2 groups: 20 patients in the mirror group, and 20 patients in the control group. All patients completed the study. Fig. 1 shows the study flowchart.

The demographic and clinical features of the patients are presented in Table I. Mirror and control groups were similar regarding demographic and clinical variables. All patients had clinical signs of acute stage of CRPS, with duration of pain less than 3 months in all patients. The CRPS-1 aetiology was mostly distal radius fracture in both groups (Table I).

**Table I:** Sociodemographic and clinical features of the patients

Factor	Mirror group	Control group	*p*-value
Age, years, mean (SD)	52.55 (16.21)	50.85 (18.81)	0.761
Gender, *n* (%)			0.723
Male	6 (30.0)	5 (25.0)	
Female	14 (70.0)	15 (75.0)	
Job, *n* (%)			0.870
Housewife	11 (55.0)	10 (50.0)	
Employee	5 (25.0)	5 (25.0)	
Officer	1 (5.0)	3 (15.0)	
Retired	3 (15.0)	2 (10.0)	
Dominant hand, right, *n* (%)	20 (100.0)	19 (95.0)	1.000
Time since injury, day, mean (SD) Median (IQR)	71.00 (49.11) 60.0 (29.8)	56.05 (17.09) 57.5 (26.3)	0.289
Duration of pain, day, mean (SD) Median (IQR)	38.1 (16.75) 35.0 (14.0)	38.5 (15.84) 35.0 (22.5)	0.967
History of immobilization, *n* (%)	19 (95.0)	20 (100.0)	1.000
Affected hand, *n* (%)			0.342
Right	9 (45.0)	12 (60.0)	
Aetiology, *n* (%)			1.000
Distal radius fracture	12 (60.0)	13 (65.0)	
Other upper extremity fracture	5 (25.0)	5 (25.0)	
Other^a^	3 (15.0)	2 (10.0)	
History of operation, *n* (%)	13 (65.0)	8 (40.0)	0.113
Pregabalin use, *n* (%)	5 (25.0)	7 (35.0)	0.490

a TFCC repair, dorsal radiocarpal ligament repair, rotator cuff tendon repair, chronic extensor tendon repair.

SD: standard deviation; IQR: interquartile range; TFCC: triangular fibrocartilage complex.

The change in outcome variables by treatment for each group is presented in Table II. There were statistically significant improvements in both groups after the treatment at both time points in terms of pain, grip and pinch strength, hand circumference at the wrist, hand dexterity, and hand activities. In the control group, hand circumference measurements at the MCP joint level did not show a statistically significant change after the therapy (*p* > 0.05). Regarding the sub-sections of NHP, pain, emotional reactions, and sleep scores revealed significant improvements in the mirror group at follow-up only, whereas in the control group only sleep scores at follow-up improved significantly.

**Table II:** Change of outcome variables by treatment compared with baseline in each group

Variables	Mirror group			Control group		
	Mean (SD)	Median (IQR)	*p*-value*	Mean (SD)	Median (IQR)	*p*-value*
Pain (0–10 NRS)						
Baseline	5.15 (2.23)	5.5 (8.0)		6.10 (2.71)	7.0 (3.8)	
After (4th week)	2.95 (2.06)	3.0 (4.0)	**0.002**	3.65 (2.66)	3.0 (2.8)	**< 0.001**
Follow-up (8th week)	2.25 (2.38)	1.5 (4.8)	**0.002**	4.10 (2.92)	5.0 (6.3)	**0.038**
Grip strength (kg)						
Baseline	5.48 (5.12)	4.0 (6.3)		6.53 (4.66)	5.0 (4.8)	
After (4th week)	11.25 (5.98)	10.5 (10.8)	**< 0.001**	12.95 (7.81)	10.5 (7.8)	**< 0.001**
Follow-up (8th week)	15.60 (10.15)	12.2 (15.3)	**< 0.001**	15.80 (7.93)	14.0 (7.8)	**< 0.001**
Pinch strength (kg)						
Baseline	2.88 (1.86)	2.2 (2.9)		3.26 (1.89)	3.0 (2.0)	
After (4th week)	4.69 (1.66)	4.5 (2.5)	**< 0.001**	4.83 (2.20)	4.5 (2.4)	**< 0.001**
Follow-up (8th week)	6.19 (2.35)	6.0 (4.0)	**< 0.001**	5.58 (1.73)	5.5 (2.4)	**< 0.001**
Wrist circumference (cm)						
Baseline	17.85 (1.25)	18.0 (1.8)		17.75 (1.39)	17.2 (1.0)	
After (4th week)	17.30 (1.28)	17.0 (1.0)	**0.002**	17.30 (1.28)	17.0 (1.8)	**0.004**
Follow-up (8th week)	17.03 (1.22)	16.7 (1.5)	**< 0.001**	17.15 (1.36)	17.0 (1.5)	**0.022**
MCP 3rd circumference (cm)						
Baseline	19.85 (1.28)	19.5 (1.9)		19.88 (1.69)	19.5 (2.0)	
After (4th week)	19.43 (1.13)	19.0 (1.5)	**0.008**	19.65 (1.68)	19.2 (2.9)	0.060
Follow-up (8th week)	19.43 (1.16)	19.2 (1.5)	**0.014**	19.78 (1.69)	19.2 (2.5)	1.000
MPUT (second)						
Baseline	58.08 (62.21)	28.0 (70.8)		34.10 (34.27)	19.2 (23.3)	
After (4th week)	20.78 (11.34)	17.5 (8.8)	**< 0.001**	18.90 (9.34)	18.0 (7.0)	**0.002**
Follow-up (8th week)	16.99 (5.32)	15.5 (5.5)	**< 0.001**	16.15 (5.23)	16.0 (8.1)	**< 0.001**
DHI (0–90)						
Baseline	54.45 (23.13)	51.0 (43.5)		52.95 (23.46)	58.0 (38.8)	
After (4th week)	28.00 (15.82)	29.0 (21.8)	**< 0.001**	26.55 (17.58)	22.0 (28.5)	**< 0.001**
Follow-up (8th week)	14.15 (12.22)	12.5 (14.3)	**< 0.001**	20.65 (15.59)	18.0 (25.8)	**< 0.001**
NHP-Pain (0–100)						
Baseline	35.63 (30.42)	37.5 (56.3)		46.88 (36.92)	50.0 (62.5)	
After (4th week)	28.75 (32.72)	18.7 (56.3)	0.610	31.25 (31.80)	18.7 (50.0)	0.098
Follow-up (8th week)	16.88 (25.42)	6.2 (34.4)	**0.016**	37.50 (30.35)	37.5 (59.4)	0.058
NHP-Emotional reactions (0–100)						
Baseline	31.11 (33.55)	22.2 (52.8)		43.33 (30.99)	50.0 (52.8)	
After (4th week)	23.33 (36.21)	0.0 (50.0)	0.078	35.55 (28.29)	33.3 (41.7)	0.908
Follow-up (8th week)	13.33 (18.94)	0.0 (22.2)	**0.010**	26.11 (22.88)	27.8 (30.6)	0.070
NHP-Sleep (0–100)						
Baseline	36.67 (35.11)	20.0 (80.0)		51.00 (27.10)	50.0 (55.0)	
After (4th week)	30.00 (31.46)	20.0 (55.0)	0.374	40.00 (33.09)	40.0 (70.0)	0.076
Follow-up (8th week)	16.00 (23.93)	0.0 (20.0)	**0.048**	38.00 (34.27)	40.0 (75.0)	**0.014**
NHP-Social isolation (0–100)						
Baseline	23.00 (31.30)	10.0 (50.0)		25.00 (28.19)	20.0 (40.0)	
After (4th week)	18.00 (29.66)	0.0 (20.0)	0.602	16.00 (24.79)	0.0 (20.0)	0.194
Follow-up (8th week)	14.00 (20.62)	0.0 (40.0)	0.140	9.00 (17.74)	0.0 (15.0)	0.058

SD: standard deviation; IQR: interquartile range; NRS: numeric rating scale; MPUT: Moberg Pick-up Test; DHI: Duruöz Hand Index; NHP: Nottingham Health Profile.

* Bonferroni adjustment was applied; bold *p*-values indicate statistical significance.

When the groups were compared regarding the improvements in outcome variables (change scores), no statistically significant difference was found between the 2 groups on any of the outcomes (*p* > 0.05) (Table III).

**Table III:** Between-group comparison of outcome variables by treatment

Variables	Mirror group Change score (Δ)		Control group Change score (Δ)		P*
	Mean (SD)	Median (IQR)	Mean (SD)	Median (IQR)	
Pain (0–10 NRS)					
Baseline–After	–2.20 (2.12)	–2.0 (2.8)	–2.45 (2.24)	–2.0 (3.0)	1.000
Baseline–Follow-up	–2.90 (2.59)	–3.0 (4.5)	–2.00 (3.55)	–3.0 (6.8)	1.000
Grip strength (kg)					
Baseline–After	5.78 (3.20)	5.0 (4.8)	6.43 (5.50)	5.0 (5.0)	1.000
Baseline–Follow-up	10.13 (5.89)	8.5 (10.0)	9.28 (4.74)	8.2 (5.4)	1.000
Pinch strength (kg)					
Baseline–After	1.81 (0.96)	1.7 (1.5)	1.56 (1.04)	1.5 (1.6)	0.690
Baseline–Follow-up	3.31 (1.33)	3.5 (1.9)	2.31 (1.11)	2.6 (1.8)	0.050
Wrist circumference (cm)					
Baseline–After	–0.55 (0.54)	–0.5 (1.0)	–0.45 (0.43)	–0.5 (1.0)	1.000
Baseline–Follow-up	–0.83 (0.69)	–0.7 (0.5)	–0.60 (0.85)	–1.0 (0.5)	1.000
MCP 3^rd^ circumference (cm)					
Baseline–After	–0.43 (0.52)	–0.5 (1.0)	–0.23 (0.41)	0.0 (0.5)	0.396
Baseline–Follow-up	–0.4380.65)	–0.5 (0.5)	–0.10 (0.64)	0.0 (0.9)	0.120
MPUT (second)					
Baseline–After	–37.30 (57.80)	–10.0 (34.9)	–15.20 (31.30)	–5.2 (14.9)	0.100
Baseline–Follow-up	–41.09 (61.45)	–11.5 (59.8)	–17.95 (31.82)	–6.5 (14.8)	0.280
DHI (0–90)					
Baseline–After	–26.45 (19.78)	–28.0 (21.3)	–26.40 (20.34)	–22.0 (20.3)	1.000
Baseline–Follow-up	–40.30 (17.67)	–44.0 (27.0)	–32.30 (24.48)	–29.5 (29.8)	0.582
NHP-Pain (0–100)					
Baseline–After	–6.88 (32.06)	0.0 (25.0)	–15.63 (31.64)	–6.25 (34.4)	0.648
Baseline–Follow-up	–18.75 (26.44)	–12.5 (37.5)	–9.38 (17.15)	–12.5 (25.0)	0.470
NHP-Emotional reactions (0–100)					
Baseline–After	–7.78 (15.34)	0.0 (19.4)	–7.78 (35.89)	5.6 (52.8)	1.000
Baseline–Follow-up	–17.78 (22.91)	–22.2 (22.2)	–17.22 (33.33)	0.0 (50.0)	1.000
NHP-Sleep (0–100)					
Baseline–After	–6.67 (20.63)	0.0 (20.0)	–11.00 (21.98)	0.0 (20.0)	1.000
Baseline–Follow-up	–20.67 (38.69)	–20.0 (55.0)	–13.00 (17.50)	–20.0 (20.0)	1.000
NHP-Social isolation (0–100)					
Baseline–After	–5.00 (20.39)	0.0 (15.0)	–9.00 (32.75)	–10.0 (20.0)	0.668
Baseline–Follow-up	–9.00 (21.00)	0.0 (20.0)	–16.00 (30.85)	–20.0 (20.0)	0.608

SD: standard deviation; IQR: interquartile range; NRS: numeric rating scale; MPUT: Moberg Pick-up Test; DHI: Duruöz Hand Index; NHP: Nottingham Health Profile.

* Bonferroni adjustment was applied.

## DISCUSSION

The results of this study revealed that mirror therapy applied in addition to routine physical therapy and a rehabilitation programme did not provide extra benefit for the improvement of clinical outcomes in patients with acute post-traumatic CRPS-1 of the hand. This is the first randomized controlled study investigating the effects of mirror therapy on pain, function, and other clinical outcomes in post-traumatic CRPS-1. The effectiveness of isolated mirror therapy in the management of trauma-induced CRPS-1 has only been studied once before in a randomized controlled trial, which solely assessed body form perception as an outcome measure and showed that mirror therapy was superior to the control group in terms of body shape perception in chronic CRPS-1 (15). However, this study did not evaluate the effects of mirror therapy on pain and function and the researchers emphasized that the effects of mirror therapy on different symptoms of CRPS should further be investigated (15).

It is thought that mirror therapy provides a reduction in pain by increasing the connection between sensory input and sensory and motor cortex, by the visual feedback from a healthy hand (5–8). This situation was first reported by Ramachandran et al. (23) in phantom pain, and it was thought that the same mechanism would apply to CRPS. In this context, mirror therapy was administered to 8 patients with CRPS-1 in a pilot study and it was found to be effective in terms of pain (14).

In the literature, there are 4 randomized controlled studies investigating the efficacy of mirror therapy in post-stroke CRPS-1 patients (10–13). Two of these studies were conducted by Cacchio et al., and mirror therapy was effective in terms of pain and motor function in patients at the subacute (mean time of CRPS 2.7 months) and chronic (> 6 months) stages of CRPS-1 (11, 12). Mirror therapy was useful in increasing the excitability of the motor cortex through sensory feedback in stroke patients, as shown by Cacchio et al. In the third study, patients in the intermediate/dystrophic stage of CRPS-1 were enrolled and mirror therapy was found to be beneficial for both pain and motor function compared with the control group (10). The researchers of this study reported that they had included patients in the subacute stage of CRPS, as spontaneous recovery could happen in the acute stage of the condition (10). The last study was conducted by Saha et al. and mirror therapy was found to be effective in terms of pain, oedema, and disability on CRPS-1 in chronic post-stroke patients (> 1 year) (13). In conclusion, all post-stroke studies indicate the effectiveness of mirror therapy for CRPS-1. It is interesting to note that, in 2 of these studies, CRPS-1 stages were subacute whereas in the third this was chronic. In the last one, CRPS-1 duration was not stated but all patients were at the post-stroke chronic phase. In our study, all patients were in the clinically acute stage of CRPS-1. The variation of our results from these studies may be due to the differences in both the duration (clinical stage) and the aetiology of CRPS-1. In connection with this, Shokouhi et al. examined the structural and functional changes in the brain in patients with early and late CRPS (24). Patients with early CRPS had decreased grey matter volume and perfusion of the somatosensory cortex and limbic system, whereas late CRPS patients showed an increase in motor cortex perfusion but no change in grey matter volume. Therefore, the anatomical alterations in the brains of those with early and late CRPS might differ. Furthermore, it has not yet been investigated whether the aetiology impacts the cortical alterations; for instance, it is unclear whether neuroimaging results in traumatic CRPS with peripheral origin are similar to those caused by a centrally elicited post-stroke CRPS.

There are also studies evaluating motor imagery therapy in patients with CRPS, in which mirror therapy is also a component. After conducting 2 randomized controlled trials, Moseley et al. concluded that mirror treatment, the final phase of the motor imagery programme, was helpful for reducing pain in people with persistent CRPS-1 (25, 26). The efficacy of mirror therapy in CRPS was evaluated in some systematic reviews recently. In the earlier paper by Méndez-Rebolledo et al., it was concluded that mirror therapy could improve pain in CRPS-1 but there was not enough evidence to recommend it over other treatments given the small size and heterogeneity of patients (5). A Cochrane review by Smart et al. (27) in 2022 concluded with low-certainty evidence that mirror therapy might provide clinically meaningful improvements in pain and function in post-stroke CRPS-1; however, its effects in post-traumatic CRPS-1 remained unknown. Another recent meta-analysis with low-quality of evidence confirmed the same results for post-stroke CRPS-1 (28). Finally, an overview of systematic reviews concluded that mirror therapy was useful for the treatment of pain and disability in only post-stroke CRPS-1 (29).

Several imaging studies have been carried out to look at alterations in the brains of CRPS patients. Maihöfner et al., using magnetoencephalography, examined the cortical changes in the acute phase of CRPS-1 developed after trauma and found a shrinkage in the area of the primary somatosensory cortex (S1) that represented the affected hand (30). Another study by Maihöfner et al. (31) examined the cortical alterations in patients with acute CRPS-1 following medication therapy. They noticed an increase in the S1 area, which shrank in the brain region that represented the affected hand. The results of this study imply that, in patients with acute CRPS, cortical abnormalities can be corrected solely by medical treatment, without the need for neurorehabilitative techniques such as mirror therapy. Mancini et al. recently investigated the alterations in the S1 by stimulating the fingertips on both hands during functional magnetic resonance imaging in 18 patients with chronic CRPS-1 and 17 healthy controls (32). Consequently, it was demonstrated that, with regard to area, location, and geometry, the affected hands of the patients in the S1 presented similarly to the hands of healthy controls and the healthy hands. The results of this study conflict with the previous imaging results. Contrary to earlier research, Mancini et al. reported that there was no decrease in S1 on the affected side in patients with CRPS and concluded that cortical sensorimotor restorative treatments, such as mirror therapy, administered to patients should be re-evaluated. They also noted that images of the earlier studies were low resolution and the measurements were indirect (32). As a result, all these imaging studies conducted in patients with CRPS do not give a clear idea regarding what kind of changes occur in the brains according to the clinical stages of CRPS and their response to treatment. It is also unclear how the reorganization processes in the brain change with different treatment strategies (e.g., medical, neurorehabilitative, interventional).

### Strengths and limitations

Our study has several strengths. First, it was designed as a randomized controlled single-blind trial. Second, to control Type 2 error statistically, a sufficient number of patients were recruited by performing power analysis and Bonferroni correction (*p* < 0.05) was applied to control Type 1 error. Finally, all of the participants shared similar aetiologies as the inciting event of the CRPS; that is, only those with trauma-induced CRPS-1 were included.

There are some limitations to the study. The first of these is the lack of neuroimaging. Second, long-term follow-up of patients after the treatment was not performed. Third, the study sample consisted of only CRPS-1 patients at the acute stage. This occurred by chance, as there was no restriction according to the clinical period of CRPS in the inclusion criteria. The inclusion of patients at the chronic stage in the sample might have been informative in terms of whether the response to mirror therapy would differ in the early vs late stages. Finally, sham mirror treatment was not applied in the control group. In sham mirror treatment, the mirror surface is covered and the patient cannot watch the reflected movements. However, as it can easily be understood by the patient that active treatment is not given in this application, how appropriate sham mirror therapy is in the context of placebo control should be discussed (33).

### Conclusion

This randomized controlled, single-blind study revealed that mirror therapy applied in addition to the routine physical therapy and rehabilitation programme in the treatment of patients with trauma-induced CRPS-1 did not provide an additional contribution to the improvement of pain, function, and other clinical outcomes. We think that future studies with long-term follow-up, including neuroimaging and controlling the clinical stage, causative aetiology (peripheral or central), and the treatment strategy may shed more light on managing patients with CRPS-1 and perhaps provide new perspectives on treatment.
